# Altered cerebrovascular-CSF coupling in Alzheimer’s Disease measured by functional near-infrared spectroscopy

**DOI:** 10.1038/s41598-023-48965-x

**Published:** 2023-12-15

**Authors:** Hany Ferdinando, Sadegh Moradi, Vesa Korhonen, Vesa Kiviniemi, Teemu Myllylä

**Affiliations:** 1 Research Unit of Health Science and Technology, University of Oulu, Oulu, Finland; 2 Opto-Electronics and Measurement Technique Research Unit, University of Oulu, Oulu, Finland; 3 Department of Radiology, Oulu University Hospital, Oulu, Finland

**Keywords:** Neurodegenerative diseases, Dementia

## Abstract

In-vivo microscopical studies indicate that brain cerebrospinal fluid (CSF) transport driven by blood vessel pulsations is reduced in the early stages of Alzheimer’s disease (AD). We hypothesized that the coupling pattern between cerebrovascular pulsations and CSF is altered in AD, and this can be measured using multi-wavelength functional near-infrared spectroscopy (fNIRS). To study this, we quantified simultaneously cerebral hemo- and CSF hydrodynamics in early AD patients and age-matched healthy controls. Physiological pulsations were analysed in the vasomotor very low frequency (VLF 0.008–0.1 Hz), respiratory (Resp. 0.1–0.6 Hz), and cardiac (Card. 0.6–5 Hz) bands. A sliding time window cross-correlation approach was used to estimate the temporal stability of the cerebrovascular-CSF coupling. We investigated how the lag time series variation of the coupling differs between AD patients and control. The couplings involving deoxyhemoglobin (HbR) and CSF water, along with their first derivative, in the cardiac band demonstrated significant difference between AD patients and controls. Furthermore, the lag time series variation of HbR-CSF in the cardiac band provided a significant relationship, p-value = 0.04 and r^2^ = 0.16, with the mini-mental state exam (MMSE) score. In conclusion, the coupling pattern between hemodynamics and CSF is reduced in AD and it correlates with MMSE score.

## Introduction

Early diagnostics of the neurodegenerative diseases (NDDs), such as Alzheimer’s Disease (AD), are of high need. According to Alzheimer’s Disease International (ADI), 75% people with dementia are not diagnosed and it may reach up to 90% in low- and middle-income countries^1^. Since AD is a progressive NDD^2^, particularly having an early diagnosis is of highly importance. ADI also wrote that MRI or computer tomography (CT) is highly recommended in early evaluation of dementia, while positron emission tomography (PET) and single photon emission computed tomography (SPECT) are used for more complex cases. The main challenges of using these methods are radiation safety, requirements of educated personal to use the imaging technology and high invest and maintenance costs, limiting their wider usage. Nevertheless, functional neuroimaging offers a great potential for early diagnostics of NDDs as it may possibly detect deviation in neuronal activities before neuronal damage^3^.

Functional near infrared spectroscopy (fNIRS) is commonly used for measuring human cerebral hemodynamics, e.g., to decipher the coupling between neural activity and cerebral blood flow (CBF) controlled by the neurovascular unit (NVU). The fNIRS technology development in recent years has made it possible to realise it also as a small, wearable brain monitoring device, making it an interesting potential alternative as a first-line brain diagnostic tool. Numerous studies show that NVU dysfunction leads to altered neurovascular coupling can be disrupted due to diseases and aging^4^. Furthermore, impaired neurovascular coupling is thought to contribute to tissue injury, loss of function, and cellular degeneration over time^5^. Interestingly, dysfunction of the neurovascular unit (NVU) controlling the coupling, has been linked with various neurodegenerative diseases (NDDs) including Alzheimer disease (AD)^6^. Impaired cerebrovascular reactivity, CBF reductions, CBF dysregulation and blood brain barrier (BBB) disfunction are now recognized to be early events in the AD pathophysiological cascade^7^.

These parameters are also linked with the glymphatic system^8^, where function of BBB, cardiovascular and cerebrospinal fluid (CSF) conduits inside the BBB driven by physiological pulsations is of high interest. In particular, the mechanism between CSF and cerebrovascular function and their coupling have awaken increased interest^9^. For instance, a very recent study by Nedergaard’s group shows that dynamic changes in vascular diameter drive perivascular glymphatic CSF inflow and this can occur in the absence of neural activation^10^. In 2019, Fultz et al., showed a strong correlation between CSF and negative of the first derivative of blood oxygen dependent level (BOLD) in a human sleep study where BOLD and CSF flow were computed by fMRI^11^. Further, Han et al. found out that the degree of correlation between BOLD and CSF was altered in AD patients compared to controls^9^. Abnormalities in cardiovascular impulse propagation with increased speed and ever reversal flow and increased BOLD signal amplitude variability have been detected in AD with fast fMRI^12,13^ in areas indicating increased BBB permeability^14^. Also, transcranial Doppler ultrasound (TCD) study showed that cerebral blood flow variability was reduced in AD despite increased blood pressure variability^15^. These findings indicate an impairment in the cerebral blood flow in rigid, narrowed and non-responsive blood vessels and suggest reduced clearance of CSF waste and metabolites that predisposes to neurodegeneration (NDD) and cognitive decline.

In addition to fMRI, fNIRS shows potential for detecting hemodynamic abnormalities in patients with AD performing controlled tasks. For instance, Li et al. found differences in hemodynamic response in frontal lobe when doing digit verbal span task^16^, and Arai et al. monitored the hemodynamic response from frontal, left and right parietal, and occipital areas when employing verbal fluency task^17^. They found that controls had higher activation index than AD patients based on the amplitude changes in the oxyhemoglobin (HbO) waveform during the task. Fladby et al. showed dysfunction in temporal lobe based on olfactory response, a response due to odorous substances detection^18^. As a complement to studies using fMRI, Zeller et al. utilised fNIRS to measure both HbO and deoxyhemoglobin (HbR) from midline parietal (Pz) area from AD patients and controls while doing modified Benton Line Orientation Task^3^, and found fNIRS as a promising tool to separate AD patients from controls. Furthermore, it seems that cerebral hemodynamics in AD is different compared to healthy controls also in resting state, as it was shown by Ferdinando et al.^19^.

Myllylä et al. introduced fNIRS based method to measure non-invasively in human brain water variations in subarachnoid space reflecting CSF dynamics and validated it in fMRI^20^. Furthermore, our fNIRS method showed that vascular fluctuations of cerebral blood and brain water concentrations have high anticorrelation^21,22^. One particular advantage of the fNIRS technique is its ability to simultaneously measure the cerebral hydrodynamics of HbO, HbR, total hemoglobin (HbT) and CSF with high temporal resolution enabling possibility to effectively study the temporal dynamics and stability of the hydrodynamic couplings. In this present study, we explored the temporal stability of the coupling pattern between the hemodynamic vs. CSF in AD patients and age-matched controls. We found that the hydrodynamic coupling between the brain blood and CSF signal was altered in the cardiac band.

## Results

**Figure 1:**
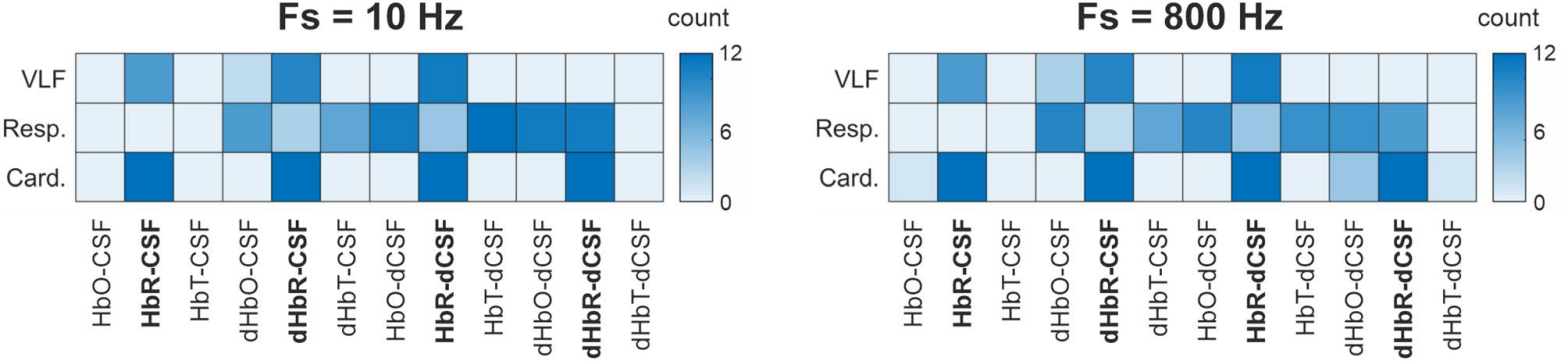
The heat maps from 10 and 800 Hz data summarise results from this study. The heat maps revealed that SD of coupling pattern from cardiac band involving HbR and its first derivate (dHbR) provided good separation between AD patients and controls, see the bold face pairs. Results from the VLF and respiratory bands from both sampling frequencies looked slightly different.

In this study we analysed temporal stability of the coupling pattern between cortical brain blood signals vs. CSF water signals over time in different time windows and overlaps. Based on the CSF water vs. blood (HbO, HbR, and HbT) cross-correlation analysis, see supplementary Tables S1–S4, we counted the number of significant differences between AD patients and controls based on p = 0.05 significant level to create a heatmap to summarise results for each band of interest, see Fig. 1. The colour bars on the right of each heatmap were scaled from zero to twelve, representing twelve combinations from four window width values and three overlap parameters. These heatmaps revealed that the cardiac band provided promising results.

**Figure 2:**
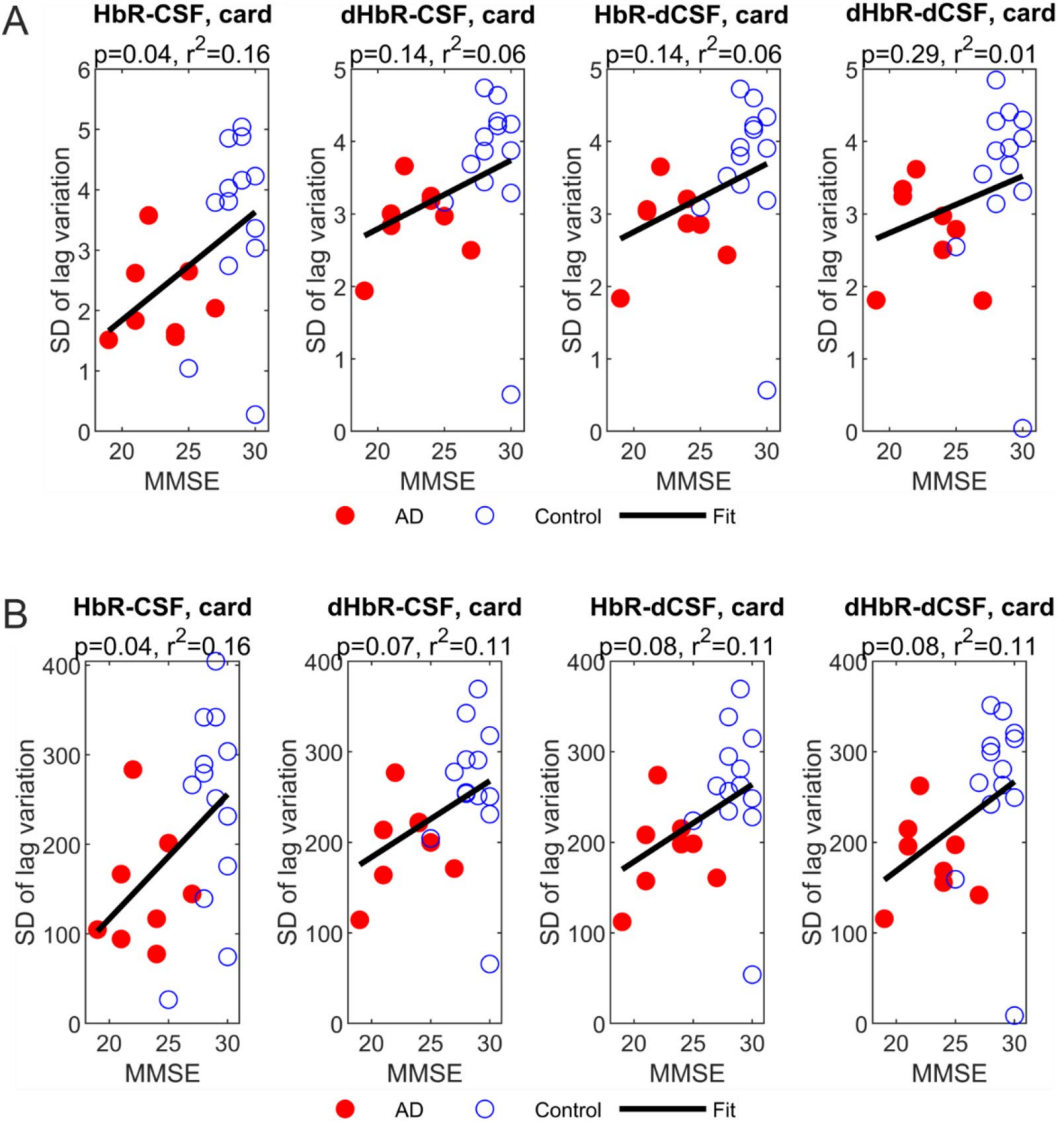
Scatter plots of MMSE vs. SD of lag variation analysis with the fitting linear line (**A**) cardiac band at 10 Hz, (**B**) cardiac band at 800 Hz. Red dots present AD patients and blue rings healthy controls.

We also conducted a regression analysis of mini-mental state examination (MMSE) score against these variations, see Fig. 2. All plots present similar pattern with positive gradient pairs. In the cardiac band, both 10 Hz and 800 Hz sampling frequencies achieved good results in HbR-CSF coupling (p-val < 0.05 and r^2^ = 0.16). Figure 2 also demonstrates that the time lag variation was in agreement with MMSE score.

## Discussions

We studied hemodynamic–CSF coupling pattern in AD patients and age-matched controls using novel multiwavelength fNIRS method. We applied a sliding window cross-correlation analysis to quantify the coupling pattern between cortical brain hemodynamics and CSF signals. We found that especially in the cardiac band, the controls had higher time domain lag variation in the following pair of HbR-CSF, dHbR-CSF, HbR-dCSF, and dHbR-dCSF compared to AD patients, see Tables S1–S4 in the supplementary material. Results from the pairs involving HbR and dHbR in the cardiac band stood out among the others in both 10 Hz and 800 Hz sampling frequencies. These results indicate that especially in the cardiac band the venous HbR and CSF signals in controls have more variation than those in AD patients, see supplementary Tables S1–S4.

We found that especially in the very fast with minimal overlapping window the coupling of the venous HbR blood signal and CSF signal seems to be altered into monotonous signal over time in AD. In matched healthy controls the CSF signal has more variance in its coupling pattern to the regional venous blood signal pulsations. Han et al. performed the coupling analysis using a relatively slow sampling rate of the BOLD data with TR of 2.3 s. They calculated BOLD signal by averaging the whole brain area and CSF from IV ventricle and did not use sliding window approach but rather a whole time series approach more commonly used in fMRI. They found that the overall coupling between the venous derived BOLD signal and CSF was reduced gradually from control to significant memory concern (SMC), mild cognitive impairment (MCI), and lastly AD^9^. Their finding was in concordance with our finding, that the coupling pattern variation involving the venous HbR signal provided good separation between AD patients and controls. The whole brain BOLD and CSF measured from IV ventricle may be more sensitive to T2 CSF flow changes^9,11^, while our CSF measure is more sensitive to CSF pulsations detected locally mainly from the subarachnoid space of the NIRS measurement volume. It might explain why we achieved good separation from the cardiac band (0.6–5 Hz), while Han et al. got the results within 0.01–0.1 Hz band. In our case, the sliding window approach is not as sensitive as Han et al. method for slow VLF vasomotor waves. However, the faster hydrodynamic coupling changes in the cardiac frequencies do also occur in between venous to CSF signals. Furthermore, these variations reflect significant relationships with MMSE (p-value = 0.04, r^2^ = 0.16), see also Fig. 2, from HbR-CSF in both 10 Hz sampled data that can be achieved with MREG scanning^12^, as well as well as with the fast 800 Hz sampling frequency. They both seemed providing good reflection for cognitive capability as well. However, larger number of subjects must be studied to confirm this. Future work includes data collection from more subjects and study the correlation with the mild, moderate, and severe state of AD.

In our data the variation in AD patients were always smaller than that in controls. The AD patients suffer from vascular wall pathology^12^, linked to brain tissue atrophy^23^ that leads to stiffening brain tissue^24^. Stiffened tissue and blood vessels may fail to both pulsate and drive CSF along perivascular spaces. Furthermore, stiffened arterioles fail to respond to local demands of activation hyperremia that is normally dynamically ongoing as driven by neurovascular coupling^12,15^. In other words, the damaged arterioles seem to only convey monotonic flow pulsations that fail respond to rapid neurovascular control cues. The reduced dynamics in the coupling between the HbR and CSF signals may further reflect reduced dynamics in the blood flow drive of CSF.

From the three physiological bands involved in the analysis, the cardiac band showed promising results with all window width and overlap parameters combination to separate AD patients from controls, see heatmaps in Fig. 1 and Tables S1–S4 in the Supplementary materials section. With the narrowest window width and the largest overlap, analysis in the cardiac band results around 130 samples for cross-correlation operation. It means there were around 130 lag-values stored for statistical analysis. On the contrary, both the VLF and respiratory bands only provided less than 10 and 30 samples respectively using the same scenario. One possible reason was the number of values included in the statistical analysis was important. We also conducted analysis with the whole signal without using a sliding window and we did not get any significant differences between AD patients and controls.

To validate this hypothesis, we conducted further experiments on the cardiac band by reducing the signal length up to 25% of the original one to mimic the situation in the respiratory band. It meant the signal of interest only lasted for 1.25 min. The length of the sample reduced drastically and even the lowest number of samples among all window width and overlap parameter was about 30. Interestingly, only HbR-CSF and dHbR-dCSF pair showed their consistencies with the same number of hits. When the same procedure was applied to fit to mimic situation in the VLF band, we got similar results as in the respiratory band. Thus, the length of the sample must be considered carefully. Looking at the results from the VLF band, see Fig. 1A, some of the results from the pairs involving HbR and dHbR showed significant difference but not as many as in the cardiac band. However, signals in the VLF band were not that long for this analysis model.

There is a general agreement that CSF flow is driven by physiological signals generated by heart function, breathing, body/muscle motions, and vasomotor activity. Interestingly, the cerebrovascular-CSF coupling, and their pulsations measured in macro scale by the presented NIRS technique seem to reflect these interactions. Aging changes the mechanical properties of brain and vessel tissue stiffness, however, in AD patients these changes are more significant, and seems to affect the cerebrovascular-CSF coupling. Furthermore, our results indicate that the coupling is more drastically altered associated with the cardiac activity.

We envisage that fNIRS could be used in near future as a supportive technique to assess changes in fast brain temporal hydrodynamics in AD, while more detailed diagnosis is performed using the golden standard. The fNIRS is portable, easy to use non-invasive measure of that offers a good tool for analysing temporal dynamics due to the fast sampling of brain hydrodynamics. FNIRS can be easily used also outside hospital enabling a possibility to monitor the brain condition and progress of AD more frequently for a large aging population.

## Materials and methods

### Measurement protocol

The data collection followed the guidelines established by the Declaration of Helsinki and all participants signed informed consent letter prior to the measurements. All measurements took place in Oulu University Hospital, Oulu, Finland. This study was approved by the regional medical research ethics committee of the Wellbeing services county of North Ostrobothnia. A sample of 8 patients (6 females, 62.6 ± 2.1 year-old) with AD and 14 age-matched controls (6 females, 63.6 ± 2.9 year-old) participated in this study, who received detailed explanation before signing the consent letter. Most of the participants also filled the mini-mental state exam (MMSE) prior to the measurements. Based on the MMSE score, 22.8 ± 1.6 and 28.5 ± 1.5 for AD and controls respectively, AD patients were categorized in a mild dementia^19^.

During measurement, all participants were in a supine position in the fast fMRI chamber (10 Hz) and multiwavelength fNIRS probes, 690, 810, 830, and 980 nm, were placed on the left side of the forehead. The light source was in the middle of the forehead, while the light detector was 3 cm away from the source. These optodes were placed about 3 cm above the eyebrow. We measured using both fNIRS and fMRI simultaneously for 5 min, and the participants were instructed to simply lay still comfortably and focus on the visual screen cross in resting state^25^.

### fNIRS device

The fNIRS device utilizes frequency-coding technique to label different wavelengths, highly sensitive detection and lock-in amplification technique with a very narrow bandwidth filter (1–10 mHz) in the receiver to minimize noise^26^. MRI compatible optical fibres used in the NIRS device were fabricated by Schott and then customized for the application by Fiberoptics Technology. Having a refractive index of approximately 1.58 and an internal transmittance of 0.999 in the range of 600–1060 nm, the fibres can guide 4 light sources to 1 fibre output. With a diameter of 2.5 mm and a length of 10 mm, the tip of the output has a 90° bend and is attached to the head by a plastic fibre clip. All fibres have the same physical dimensions and measure of 10 m in length to allow combining NIRS measurements with MRI, such that the NIRS device can be placed outside the MRI room with only the optodes and fibres brought inside the room through a waveguide in the wall. This arrangement makes it possible to ensure the MRI compatibility of the NIRS measurement. Our fNIRS device uses wavelengths of 690, 810, 830 and 980 nm produced by high-power light-emitting diodes, each modulated at a specific frequency and then demodulated in the receiver amplifier. The wavelength of 980 nm is selected to ensure high sensitivity for water (CSF) dynamics, since it is strongly absorbed by water^20^. Sufficient penetration depth to measure brain cortex and subarachnoid space were ensured by using 3 cm source-detector distance, based on light propagation studies in the brain^27,28^.

Our fNIRS device sampled the measurement at 800 Hz and it was developed for other studies as well, which requires high temporal resolution, e.g., pulse shape analysis. We employed modified Beer-Lambert law (MBLL) to calculate concentration changes of HbO, HbR, and water with the following extinction coefficients: 690 nm: 0.312, 2.1392, 0.0205; 830 nm: 1.0507, 0.7804, 0.1459; 980 nm: 1.2513, 0.4233, 2.1491, and differential path length factor of 5.93. Total hemoglobin (HbT) is the sum of HbO and HbR. The concentration of CSF water was quantified based on the method presented in paper^20,29^.

### Signal analysis

**Figure 3:**
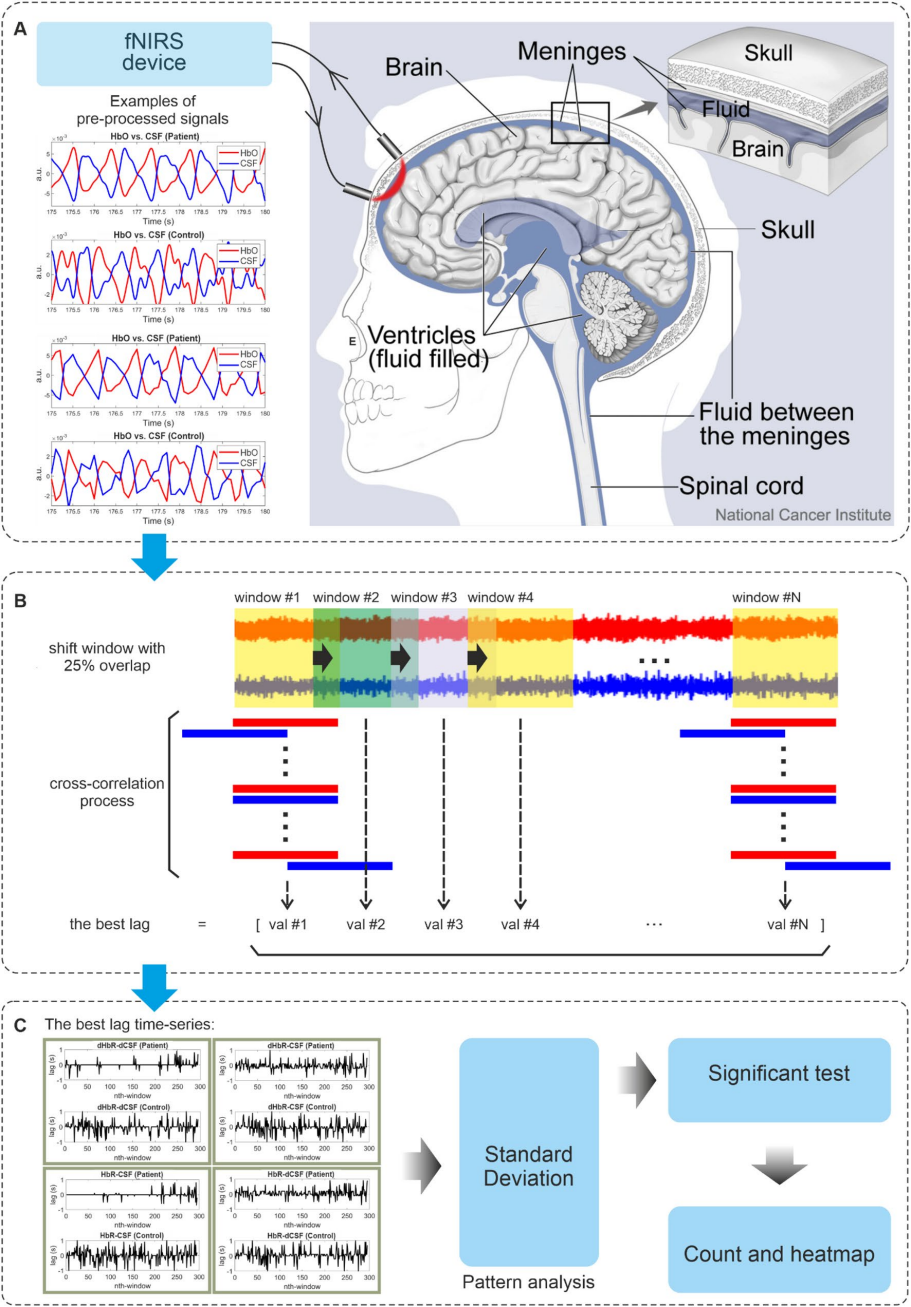
We measured fNIRS signals (CSF water, oxygenated HbO and deoxygenated HbR blood) from the forehead area using a multiwavelength fNIRS able to sense water concentration (**A**). Sample signals in the cardiac band taken from the same subject within the same time frame were given. Signal analysis model utilises cross-correlation process for each pair within pre-defined time window, red and blue represent hemodynamic and CSF signals respectively (**B**). The window is shifted with certain overlap. This diagram represents 25% overlap of the window. For cross-correlation process in each window we tracked the lag value achieving the best correlation coefficient. Collecting these lag values across the whole signals forms a series that is subjected to pattern analysis using standard deviation (SD) (**C**). Finally, a significant test using Wilcoxon-test at 0.05 significant level was used.

The analysis was conducted in the very low frequency or VLF (0.008–0.1 Hz), respiratory (0.1–0.6 Hz), and cardiac (0.6–5 Hz) bands. We analysed these coupling relationships in the original sampling and downsampling frequencies, 800 Hz and 10 Hz, respectively. Downsampling from 800 to 10 Hz enabled more comprehensive analysis with MREG data in the future. A sliding window with pre-defined width for each band was set. The window width was determined based on the maximum wavelength of the targeted physiological signal source in the selected bands; in the VLF vasomotor band the window was set to 120 s, in the respiratory band for 10 s, and 2 s for the cardiac band^21^. We extended this width up to 1.5 times of the starting width with two values in between to get four window width values for each band. For example, the window width parameters in the VLF band were 120, 140, 160, and 180 s. Using a larger window, we should be able to analyse signal more than one period of oscillation. We used 25%, 50%, and 75% overlap when the window was shifted, see Fig. 3B for 25% overlap. Therefore, we had twelve scenarios for signal analysis. The main goal of using these scenarios was to evaluate the consistency of the results.

Similar to preceding BOLD studies^9,11^, we were interested to find out whether we could analyse the hemodynamic-CSF coupling with our fNIRS system and whether these couplings were different between AD patients and controls. For this reason, we analysed the following measured signal pairs: HbO-CSF, HbR-CSF, and HbT-CSF. Motivated by the works of Han et al.^9^ to use include signal amplitude change speed in time-domain by both fMRI BOLD, we also used the first derivative of fNIRS signals. Hence, the following pairs: dHbO-CSF, dHbR-CSF, dHbT-CSF, HbO-dCSF, HbR-dCSF, HbT-dCSF, dHbO-dCSF, dHbR-dCSF, and dHbT-dCSF, where the prefix ‘d’ denotes the first derivative of the corresponding signals, were used.

These pairs were subjected to cross-correlation operation within a pre-defined sliding window, see Fig. 3B. The maximum lag of this operation was set to half of the window width. Within each window, we tracked a lag value with the best correlation coefficient, i.e., val #1, val #2, etc., see Fig. 3B. The lag value with the best correlation coefficient can be considered as a simple phase shift analysis. The lag values form a time series signal, see Fig. 3C, which bring such a pattern to characterise AD patient and controls.

From the samples of the lag time series in Fig. 3C, we could immediately see that AD patients had less variation than controls. This led to use standard deviation (SD) as a simple pattern analysis tool to characterize these two groups.

Based on Kolmogorov–Smirnov method, we found that data distribution from each group was not normal. Moreover, since the number of subjects participated in this study was small, we utilised non-parametric test, i.e., Wilcoxon-test, to evaluate significant difference between AD patients and control at 0.05 significant difference.

### Supplementary Information

Supplementary Tables.

## Data Availability

The datasets generated and/or analysed during the current study are not publicly available because we have no permission to share raw data but are available on reasonable request. Please contact Teemu Myllylä (teemu.myllyla@oulu.fi) for this request.

## Abbreviations

AD: Alzheimer's disease
Card.: Cardiac band (0.6–5 Hz)
CSF: Cerebrospinal fluid
CTRL: Control
dHbO: The first derivative of oxygenated hemoglobin
dHbR: The first derivative of deoxygenated hemoglobin
dHbT: The first derivative of total hemoglobin
dCSF: The first derivative of cerebrospinal fluid
Fs: Sampling frequency in Hz
HbO: Oxygenated hemoglobin
HbR: Deoxygenated hemoglobin
HbT: Total hemoglobin
MMSE: Mini mental state exam
Resp.: Respiratory band (0.1–0.6 Hz)
SD: Standard deviation
val: Value
VLF: Very low frequency band (0.008–0.1 Hz)
