# Supplementary material for: Altered cerebrovascular-CSF coupling in Alzheimer’s Disease measured by functional near-infrared spectroscopy
Source: Sci Rep. 2023 Dec 15;13:22364. doi: 10.1038/s41598-023-48965-x (PMC10724150; doi:10.1038/s41598-023-48965-x)
Supplement: Supplementary file 1 — Supplementary Tables. [file 41598_2023_48965_MOESM1_ESM.pdf]

Supplementary materials

Table S1. Compare SD based on the lag value series from HbR-CSF coupling in cardiac band

Window width (s)	Overlap (%)	Sampling frequency = 10 Hz			Sampling frequency = 800 Hz		
		AD Patients (s)	Controls (s)	p-value	AD Patients (s)	Controls (s)	p-value
2.0	25	0.17 ± 0.11	0.32 ± 0.14	0.0107	0.17 ± 0.10	0.31 ± 0.13	0.0107
	50	0.18 ± 0.10	0.32 ± 0.14	0.0091	0.17 ± 0.10	0.31 ± 0.14	0.0127
	75	0.17 ± 0.10	0.32 ± 0.14	0.0091	0.16 ± 0.10	0.31 ± 0.13	0.0127
2.4	25	0.19 ± 0.12	0.36 ± 0.17	0.0127	0.19 ± 0.12	0.35 ± 0.16	0.0107
	50	0.20 ± 0.12	0.36 ± 0.17	0.0127	0.20 ± 0.13	0.35 ± 0.16	0.0174
	75	0.20 ± 0.12	0.35 ± 0.17	0.0127	0.18 ± 0.12	0.35 ± 0.16	0.0174
2.6	25	0.21 ± 0.13	0.38 ± 0.17	0.0149	0.20 ± 0.13	0.38 ± 0.18	0.0107
	50	0.21 ± 0.13	0.37 ± 0.17	0.0127	0.20 ± 0.13	0.38 ± 0.17	0.0091
	75	0.22 ± 0.12	0.37 ± 0.17	0.0127	0.19 ± 0.13	0.38 ± 0.18	0.0127
3.0	25	0.22 ± 0.13	0.40 ± 0.19	0.0149	0.21 ± 0.15	0.41 ± 0.20	0.0127
	50	0.22 ± 0.13	0.39 ± 0.20	0.0174	0.21 ± 0.15	0.42 ± 0.20	0.0149
	75	0.22 ± 0.12	0.39 ± 0.19	0.0107	0.22 ± 0.16	0.41 ± 0.21	0.0127

AD = Alzheimer's disease

Table S2. Compare SD based on the lag value series from dHbR-CSF coupling in cardiac band

Window width (s)	Overlap (%)	Sampling frequency = 10 Hz			Sampling frequency = 800 Hz		
		AD Patients (s)	Controls (s)	p-value	AD Patients (s)	Controls (s)	p-value
2.0	25	0.23 ± 0.09	0.35 ± 0.10	0.0017	0.23 ± 0.08	0.34 ± 0.10	0.0021
	50	0.23 ± 0.09	0.35 ± 0.10	0.0014	0.23 ± 0.08	0.34 ± 0.10	0.0021
	75	0.23 ± 0.09	0.35 ± 0.10	0.0017	0.23 ± 0.08	0.33 ± 0.10	0.0025
2.4	25	0.26 ± 0.10	0.38 ± 0.12	0.0014	0.25 ± 0.10	0.37 ± 0.12	0.0031
	50	0.26 ± 0.10	0.38 ± 0.12	0.0021	0.25 ± 0.11	0.37 ± 0.12	0.0037
	75	0.26 ± 0.09	0.38 ± 0.12	0.0014	0.25 ± 0.10	0.38 ± 0.12	0.0031
2.6	25	0.27 ± 0.10	0.40 ± 0.13	0.0037	0.26 ± 0.10	0.39 ± 0.13	0.0045
	50	0.27 ± 0.10	0.40 ± 0.12	0.0021	0.26 ± 0.10	0.39 ± 0.13	0.0021
	75	0.27 ± 0.10	0.40 ± 0.13	0.0021	0.26 ± 0.10	0.40 ± 0.13	0.0021
3.0	25	0.28 ± 0.10	0.42 ± 0.14	0.0025	0.27 ± 0.12	0.43 ± 0.15	0.0037
	50	0.28 ± 0.10	0.42 ± 0.14	0.0017	0.27 ± 0.12	0.43 ± 0.15	0.0045
	75	0.28 ± 0.10	0.42 ± 0.14	0.0037	0.28 ± 0.11	0.43 ± 0.15	0.0037

AD = Alzheimer's disease

Table S3. Compare SD based on the lag value series from HbR-dCSF coupling in cardiac band

Window width (s)	Overlap (%)	Sampling frequency = 10 Hz			Sampling frequency = 800 Hz		
		AD Patients (s)	Controls (s)	p-value	AD Patients (s)	Controls (s)	p-value
2.0	25	0.23 ± 0.09	0.35 ± 0.10	0.0014	0.22 ± 0.08	0.33 ± 0.09	0.0014
	50	0.24 ± 0.09	0.34 ± 0.10	0.0017	0.22 ± 0.08	0.33 ± 0.10	0.0014
	75	0.24 ± 0.09	0.34 ± 0.10	0.0021	0.22 ± 0.08	0.33 ± 0.09	0.0017
2.4	25	0.25 ± 0.10	0.39 ± 0.13	0.0021	0.24 ± 0.10	0.37 ± 0.11	0.0025
	50	0.26 ± 0.10	0.38 ± 0.13	0.0025	0.24 ± 0.11	0.37 ± 0.12	0.0025
	75	0.25 ± 0.09	0.38 ± 0.13	0.0031	0.24 ± 0.10	0.37 ± 0.12	0.0025
2.6	25	0.27 ± 0.10	0.40 ± 0.13	0.0025	0.24 ± 0.10	0.39 ± 0.13	0.0021
	50	0.27 ± 0.10	0.40 ± 0.13	0.0031	0.24 ± 0.11	0.39 ± 0.13	0.0011
	75	0.27 ± 0.10	0.40 ± 0.13	0.0045	0.24 ± 0.09	0.39 ± 0.13	0.0006
3.0	25	0.29 ± 0.11	0.42 ± 0.14	0.0037	0.27 ± 0.11	0.43 ± 0.15	0.0025
	50	0.29 ± 0.11	0.42 ± 0.15	0.0045	0.26 ± 0.11	0.43 ± 0.16	0.0031
	75	0.29 ± 0.11	0.42 ± 0.14	0.0054	0.28 ± 0.11	0.43 ± 0.15	0.0031

AD = Alzheimer's disease

Table S4. Compare SD based on the lag value series from dHbR-dCSF coupling in cardiac band

Window width (s)	Overlap (%)	Sampling frequency = 10 Hz			Sampling frequency = 800 Hz		
		AD Patients (s)	Controls (s)	p-value	AD Patients (s)	Controls (s)	p-value
2.0	25	0.20 ± 0.10	0.35 ± 0.12	0.0025	0.20 ± 0.09	0.34 ± 0.11	0.0014
	50	0.21 ± 0.10	0.35 ± 0.12	0.0021	0.21 ± 0.09	0.34 ± 0.12	0.0017
	75	0.20 ± 0.10	0.34 ± 0.12	0.0021	0.21 ± 0.10	0.34 ± 0.11	0.0014
2.4	25	0.23 ± 0.11	0.39 ± 0.14	0.0028	0.23 ± 0.11	0.38 ± 0.13	0.0021
	50	0.23 ± 0.11	0.38 ± 0.14	0.0019	0.24 ± 0.11	0.38 ± 0.14	0.0031
	75	0.23 ± 0.11	0.38 ± 0.14	0.0019	0.24 ± 0.11	0.39 ± 0.13	0.0021
2.6	25	0.24 ± 0.12	0.40 ± 0.15	0.0034	0.24 ± 0.12	0.40 ± 0/14	0.0021
	50	0.25 ± 0.12	0.40 ± 0.15	0.0034	0.25 ± 0.13	0.40 ± 0.15	0.0031
	75	0.25 ± 0.12	0.40 ± 0.15	0.0049	0.23 ± 0.12	0.39 ± 0.15	0.0054
3.0	25	0.26 ± 0.12	0.42 ± 0.16	0.0034	0.27 ± 0.13	0.44 ± 0.17	0.0031
	50	0.26 ± 0.12	0.42 ± 0.16	0.0028	0.27 ± 0.13	0.45 ± 0.17	0.0064
	75	0.26 ± 0.12	0.42 ± 0.15	0.0034	0.27 ± 0.13	0.44 ± 0.17	0.0091

AD = Alzheimer's disease
